# Using VBIM Technique to Discover ARMC4/ODAD2 as a Novel Negative Regulator of NF-κB and a New Tumor Suppressor in Colorectal Cancer

**DOI:** 10.3390/ijms23052732

**Published:** 2022-03-01

**Authors:** Matthew Martin, Rasika Mundade, Antja-Voy Hartley, Guanglong Jiang, Jiamin Jin, Steven Sun, Ahmad Safa, George Sandusky, Yunlong Liu, Tao Lu

**Affiliations:** 1 Department of Pharmacology and Toxicology, Indiana University School of Medicine, 635 Barnhill Drive, Indianapolis, IN 46202, USA; mm217@iu.edu (M.M.); rmundade1810@gmail.com (R.M.); antja-voy_hartley@dfci.harvard.edu (A.-V.H.); jinjiaminz@163.com (J.J.); wzstevensun@gmail.com (S.S.); asafa@iupui.edu (A.S.); 2 Department of Medical and Molecular Genetics, Indiana University School of Medicine, 975 West Walnut Street, Medical Research and Library Building, IB 130, Indianapolis, IN 46202, USA; ggjiang@iu.edu (G.J.); yunliu@iu.edu (Y.L.); 3 Department of Pathology and Laboratory Medicine, Indiana University School of Medicine, 340 West 10th Street, Fairbanks Hall, Suite 6200, Indianapolis, IN 46202, USA; gsandusk@iupui.edu; 4 Department of Biochemistry and Molecular Biology, Indiana University School of Medicine, 635 Barnhill Drive, Indianapolis, IN 46202, USA; 5 Indiana University Melvin and Bren Simon Comprehensive Cancer Center, Indiana University School of Medicine, 535 Barnhill Dr, Indianapolis, IN 46202, USA

**Keywords:** colorectal cancer, NF-κB, transcription factor, tumor suppressor

## Abstract

Since nuclear factor (NF) κB plays pivotal roles in inflammation and cancer, understanding its regulation holds great promise for disease therapy. Using the powerful validation-based insertional mutagenesis (VBIM) technique established by us previously, we discovered armadillo repeat-containing protein 4 (ARMC4)/outer dynein arm docking complex subunit 2 (ODAD2), a rarely studied protein known to date, as a novel negative regulator of NF-κB in colorectal cancer (CRC). High expression of ARMC4 downregulated the expression of NF-κB-dependent genes, dramatically reduced NF-κB activity, cellular proliferation, anchorage-independent growth, and migratory ability in vitro, and significantly decreased xenograft tumor growth in vivo. Co-immunoprecipitation experiments demonstrated that ARMC4 forms a complex with NF-κB. Importantly, the lower ARMC4 expression in patient tumors than normal tissues indicates its potential tumor suppressor function in CRC. Collectively, we uncovered a completely new facet of ARMC4 function by identifying it as a novel NF-κB negative regulator, thus uncovering ARMC4 as a potential new therapeutic target in CRC.

## 1. Introduction

Colorectal cancer (CRC) is a lethal disease, with about 149,500 new cases expected and about 52,980 estimated death in 2021 in the USA [1]. Therapies for CRC vary based on the severity of the disease. Surgery is used for most tumors in the early stages of CRC, whereas surgery in combination with anti-cancer drugs is used in later stages [2]. Due to high mortality rates, metastasis, recurrence, and chemoresistance in later stages of CRC, new therapeutics for CRC are urgently needed.

CRC tumorigenesis is marked by several “driver mutations” of genes encoding different tumor suppressors and promoters, including adenomatous polyposis coli (APC), p53, and Kirsten rat sarcoma viral oncogene homolog (KRAS) [3,4,5]. Among other effects, these mutations promote the activation of the nuclear factor (NF)-κB family of transcription factors, which is critical for a number of processes of tumorigenesis, including cellular proliferation, migration, and control of apoptosis [6,7].

There are five NF-κB family members in mammals (RelA (p65), RelB, c-Rel, NF-κB1 (p50), and NF-κB2 (p52)). In particular, “classical” NF-κB signaling is usually mediated by the p65/p50 heterodimer as reviewed by May et al. where it can be activated from its latent form in the cytoplasm and translocated to the nuclear to bind to κB consensus sequences in promoters to trigger target gene expression [8]. The functions of the proteins encoded by these genes frequently affect inflammation, the cell cycle, cell growth, angiogenesis, and cancer progression [9]. Unsurprisingly, hyperactivation of the NF-κB pathway is closely related to deregulation of the immune system and cancer and progression, including in CRC [3,4,10,11,12]. Thus, novel regulators of NF-κB signaling could have great potential as new therapeutic targets in CRC. In this regard, identification of novel regulators of NF-κB is of particular importance as it may open new avenues for future CRC treatment.

To identify novel therapeutic targets in the NF-κB pathway, we used a robust lentiviral validation-based insertional (VBIM) method to develop mutant cell lines that overexpress specific cellular proteins that may function as NF-κB negative regulators, thus leading to an altered cell phenotype (low NF-κB activity) that is reversible by using the Cre-lox recombination system. A full description of the VBIM technology is presented in our previous publication and has already been used to identify other regulators of NF-κB signaling [9]. In the current study, we employed this powerful method to identify armadillo repeat-containing protein 4 (ARMC4)/Outer dynein arm docking complex subunit 2 (ODAD2) as a negative regulator of NF-κB. Commonly, this gene is referred to as ARMC4, so it will be presented as ARMC4 hereafter. Prior to the work described here, ARMC4 had been identified as a theoretical therapeutic target only through genome-wide association studies (GWAS) in gastric, ovarian, and breast cancers [13,14]. Importantly, ARMC4 is a member of the ARM domain-containing superfamily, which includes APC and β-catenin, both of which are widely known to be dysregulated in CRC and can promote aberrant wingless/integrated (Wnt) pathway signaling [15]. Despite a plethora of information on other superfamily members, little is known about ARMC4’s regulation and function. Previous knowledge regarding ARMC4 is that it plays a critical role in the rare disorder primary ciliary dyskinesia (PCD) [16,17] and mouse spermatogenesis [18]. In PCD patients, multiple specific point mutations of ARMC4 cause dysfunction in its ability to bind to cilia [16,17].

We now find that ARMC4 is a novel negative regulator of NF-κB and also a potential tumor suppressor in CRC. High expression of ARMC4 downregulates the expression of NF-κB-dependent genes, many of which are cancer related. Furthermore, high ARMC4 expression in CRC cells reduces NF-κB activity, cellular proliferation, anchorage-independent growth, and migratory ability in vitro and significantly decreases xenograft tumor growth in vivo, while shARMC4 knockdown has the opposite effect. Moreover, co-immunoprecipitation (co-IP) experiments confirmed that ARMC4 forms a complex with the p65 subunit of NF-κB. Interestingly, immunohistochemistry (IHC) data showed remarkably lower ARMC4 expression levels in CRC tumors than that in normal tissues. Taken together, our data reveal that ARMC4 is a new tumor suppressor in CRC, making it an attractive novel diagnostic biomarker and therapeutic target in this disease.

## 2. Results

### 2.1. VBIM Identifies ARMC4 as a Negative Regulator of NF-κB

**Figure 1:**
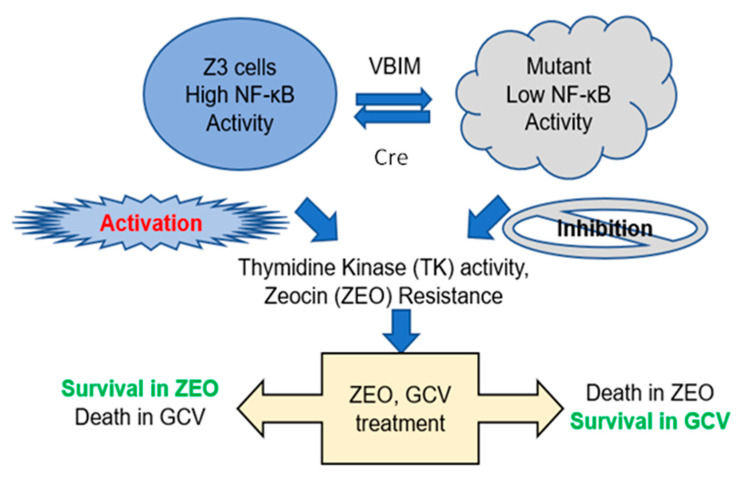
Dual drug selection system. Briefly, we start with the Z3 cells published before [9]. This is a HEK293-derived cell line with hyper nuclear factor (NF)-κB activity and engineered with NF-κB promoter-driven zeocin (ZEO) resistance gene and thymidine kinase (TK) gene. Upon infection with validation-based insertional mutagenesis (VBIM) viruses, the cells may overexpress a novel NF-κB negative regulator, therefore, changing the cell phenotype from hyper NF-κB activity to the cells with low NF-κB activity. A phenotype can be further reversed to high NF-κB activity with Cre removal of the NF-κB negative regulator gene. To select the cells with novel NF-κB negative regulator (low NF-κB activity), we will split the cells into duplicate plates, followed by the treatment with either ganciclovir (GCV), a substrate of TK, or zeocin (ZEO). As a consequence, cells with hyper NF-κB activity will survive in ZEO and die in GCV (left side), while cells harboring novel negative regulator of NF-κB, thus with low NF-κB activity, will die in ZEO and survive in GCV (right side). With this approach, a mutant cell with overexpression of a novel NF-κB negative regulator will be identified and further expanded for future experiments.

A schematic diagram for employing VBIM technology to select negative regulators of NF-κB is depicted in Figure 1. Briefly, starting with a previously published human embryonic kidney (HEK) 293-derived cell line (named Z3) with hyper NF-κB activity and NF-κB promoter-driven zeocin (ZEO) resistance gene and thymidine kinase (TK) gene [9], we employed the innovative VBIM technique to randomly overexpress an unknown NF-κB negative regulator, thus generating mutant cells with low NF-κB activity. To select out these cells (low NF-κB activity), we split the cells into duplicate plates, followed by the treatment with either ganciclovir (GCV), a substrate of TK, or zeocin (ZEO). Consequently, cells with hyper NF-κB activity survive in ZEO and die in GCV (Left side), while cells harboring novel negative regulator of NF-κB, thus with low NF-κB activity, will die in ZEO and survive in GCV (Right side). With this approach, a mutant cell with overexpression of a novel NF-κB negative regulator can be identified and further expanded for further experiments.

**Figure 2:**
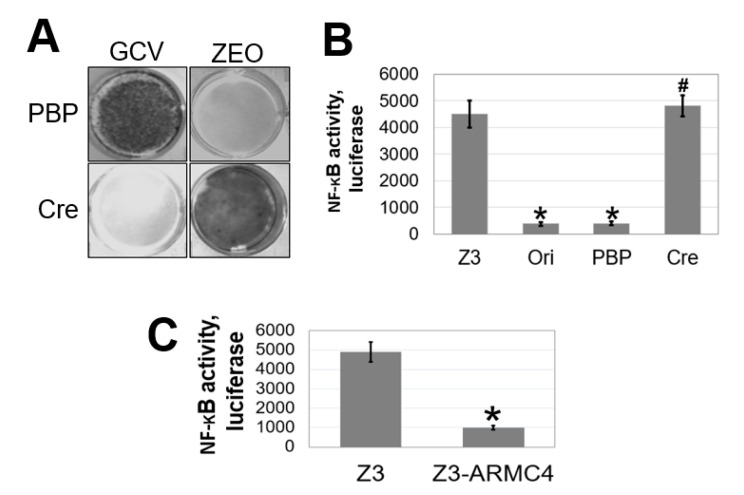
Identification of armadillo-repeat containing protein 4 (ARMC4) as a negative regulator of NF-κB using a duo-drug selection system. (**A**) Drug sensitivity of an original mutant clone selected from VBIM infected Z3 cells, as depicted from Figure 1 was further infected either with pBabe vector control (PBP) or with the expression of Cre-recombinase (Cre). A low NF-κB activity mutant with a vector PBP control died in zeocin and survived in GCV, while mutant with Cre expression had reversed phenotype. The cells died in GCV and survived in ZEO. (**B**) NF-κB luciferase assay, showing Ori mutant cells exhibited decreased NF-κB activity compared to the Z3 cells. PBP treatment exhibited similar NF-κB activity to Ori cells, which were reverted by use of Cre-recombinase to a low NF-κB activity phenotype. * *p* < 0.01 Ori and PBP vs. Z3 group; # *p* < 0.01 Cre vs. PBP group. (**C**) NF-κB luciferase assay, showing Z3 cells stably overexpressing ARMC4 have decreased NF-κB activity compared to Z3 cells. * *p* < 0.01 Z3 vs. Z3-ARMC4 group; *n* = 3.

Using this system, as shown in Figure 2A, a mutant with low NF-κB activity died in ZEO and survived in GCV, while expression of Cre expression reversed this phenotype: The cells died in GCV and survived in ZEO. We then conducted an NF-κB-specific luciferase assay in Z3 parental cells, an original mutant (Ori), or a mutant infected with the PBP control vector (PBP) or with the vector expressing Cre (Cre). As shown in Figure 2B, both Ori and PBP cells had very low NF-κB luciferase activities, while Z3 parental cells had much higher NF-κB transactivation activity. Furthermore, the low NF-κB activity in the Ori mutant was reverted by the addition of Cre-recombinase. Collectively, data from Figure 2A,B show that the gene overexpressed in the VBIM clone regulates NF-κB activity negatively.

The gene containing the VBIM vector in the Ori cells was amplified and blasted to the human genome sequence and was identified in the Ori cells as ARMC4. In Figure 2C, overexpression of ARMC4 decreased NF-κB-specific luciferase activity, affirming ARMC4’s previously unknown role as a negative regulator NF-κB.

**Figure 3:**
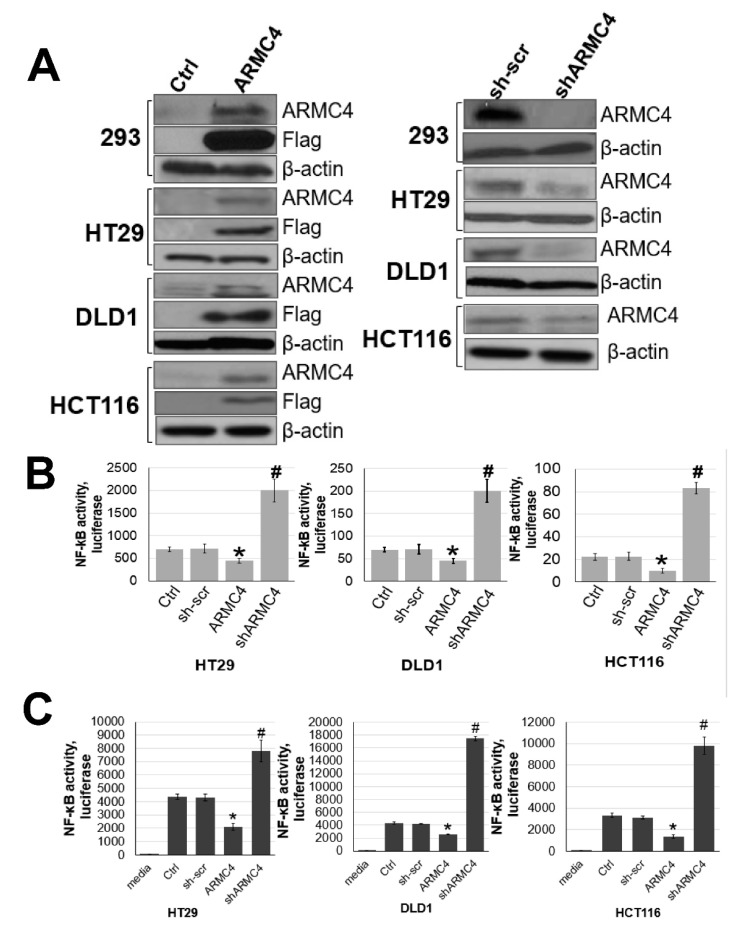
ARMC4 functions as a negative regulator of NF-κB. (**A**) Western blot, showing that Flag-tagged ARMC4 was overexpressed in CRC cell lines HT29, DLD1, and HCT116 and HEK293 cells with the vector as the control (Ctrl) (left panel) and shARMC4 stable lines were also established with the sh-scramble (sh-scr) lines as the control (right panel). (**B**) NF-κB luciferase assay, showing ARMC4 overexpression cells exhibited decreased NF-κB activity compared to the Ctrl cells while the shARMC4 exhibited increased NF-κB activity compared to the sh-scr. (**C**) Assays of conditioned media, showing that conditioned media from HT29, DLD1, and HCT116 cells overexpressing ARMC4 had much lower NF-κB activation ability than vector ctrl cells. In contrast, conditioned media from shARMC4 knockdown cells in HT29, DLD1, and HCT116 cell lines had much higher NF-κB activation ability than sh-scr cells. Stable 293-NF-κB reporter cells were used to examine the media’s NF-κB activation ability. The data were normalized to the total number of cells that generated the conditioned media and to the total amounts of protein. The data represent the means ± SD from three independent experiments. * *p* < 0.05 ARMC4 vs. Ctrl group; # *p* < 0.05 shARMC4 vs. sh-scr group. * *p* < 0.05 Ctrl vs. ARMC4 group; # *p* < 0.05 sh-scr vs. shARMC4 group; *n* = 3.

Because of these findings, we decided to further examine the functional effects of ARMC4 expression in CRC as many cancers, including CRC, are known to have constitutive NF-κB activity [5]. As shown in Figure 3A, human ARMC4 protein was either overexpressed or knocked down using shRNA in HT29, DLD1, and HCT116 CRC cells. Western blot was conducted to confirm their phenotype. Overexpression and knockdown lines were also confirmed in HEK293 cells. By using these panels of cells, we performed an NF-κB luciferase activity assay to determine ARMC4’s effects on NF-κB activity (Figure 3B). High ARMC4 expression in our CRC cell line models decreased NF-κB activity compared to the control cells, while low expression of ARMC4 from shRNA knockdown increased NF-κB activity compared to the sh-scramble control. Additionally, ARMC4 expression levels in overexpression and knockdown lines altered the microenvironment of cancer cells in a conditioned media assay, as shown in Figure 3C. Media from cells with high ARMC4 expression had lower NF-κB activity induction compared to parental CRC cells media. In contrast, media from shARMC4 cells robustly increased NF-κB activation compared to the sh-scramble control cells, further suggesting that ARMC4 negatively regulated the expression of secreted factors that could activate NF-κB. This finding is consistent with the concept that activation of NF-κB enhances the production thus release of secreted factors, such as cytokines, chemokines, and growth factors, into the local microenvironment or cell culture media of CRC cells. 

### 2.2. ARMC4 Downregulates NF-κB Target Gene Expression and Regulates Multiple Gene Networks

**Figure 4:**
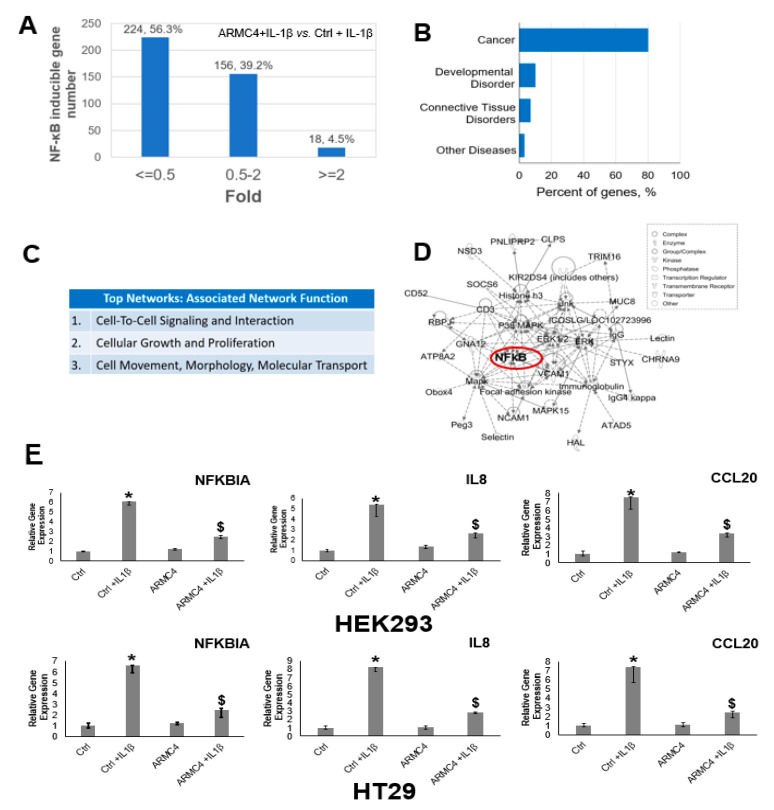
ARMC4 expression decreased NF-κB target gene expression. (**A**) An RNA-seq was conducted in the presence or absence of 10 ng/mL of IL-1β in HEK293 cells. Overexpression of ARMC4 decreased 56.3% (224 genes) of NF-κB target expression by 2-fold or more (ARMC4 + IL-1β/Ctrl + IL-1β ≤ 0.5), while leaving 39.2% (156 genes) unaffected (ARMC4 + IL-1β/Ctrl + IL-1β = 0.5–2), and merely 4.5% (18 genes) were increased by 2-fold or more (ARMC4 + IL-1β/Ctrl + IL-1β ≥ 2). Cells were treated or untreated with IL-1 β for 4 h before RNA was collected. (**B**–**D**) Ingenuity pathway analysis (IPA). IPA analysis identified near 80% of ARMC4-downregulated NF-κB target genes are cancer related (**B**), suggesting ARMC4’s important negative NF-κB regulation function in cancer. IPA also identified that ARMC4 has several top network cellular functions, including cell-cell signaling, proliferation, and cell morphology (**C**). Furthermore, IPA analysis identified a typical network that was regulated by ARMC4, in which NF-κB is at the hub of this network (**D**). (**E**) qPCR analysis of typical genes identified, confirming NFKBIA, IL-8, and CCL20 are NF-κB-inducible genes (+IL-1β condition) and were significantly downregulated by the overexpression of ARMC4 in both HEK293 (top row) and HT29 cells (bottom row) under IL-1β treatment. The data represent the means ± SD from three independent experiments. * *p* < 0.05 Ctrl + IL-1β vs. Ctrl group; $ *p* < 0.05 ARMC4 + IL-1β vs. Ctr + IL-1β group; *n* = 3.

Since we have shown ARMC4 downregulates NF-κB activity, we speculate there may be a relationship between ARMC4 expression levels and control of NF-κB-inducible gene expressions. We, therefore, conducted RNA-seq experiments to validate this hypothesis. As shown in Figure 4A, HEK293 ARMC4 overexpression cells exhibited differential gene expression compared to HEK293 controls cells. Furthermore, with addition of treatment of IL-1β, about 56.3% (224 genes) of NF-κB target genes were downregulated by two-fold or more in ARMC4 overexpressing cells (ARMC4 + IL-1β/Ctrl + IL-1β ≤ 0.5), while ~39.2% (156 genes) were not significantly affected (ARMC4 + IL-1β/Ctrl + IL-1β = 0.5–2), and only 4.5% (18 genes) were increased by two-fold or more (ARMC4 + IL-1β/Ctrl + IL-1β ≥ 2). A heatmap analysis of these genes is shown in Supplementary Figure S2. We wondered what functions these downregulated genes may be involved in. We then performed ingenuity pathway analysis (IPA) as indicated in Figure 4B, showing that a majority, ~80% of genes regulated by ARMC4, were related to cancer. IPA also suggests ARMC4 has important roles in cell signaling, cell motility, and cell growth (Figure 4C). Importantly, IPA shows NF-κB is a critical node in ARMC4-mediated signaling networks (Figure 4D). We then conducted qPCR analysis for three representative genes identified in our RNA-seq, nuclear factor of kappa light polypeptide gene enhancer in B-cells inhibitor, alpha (NFKBIA), interleukin 8 (IL-8), and C-C motif chemokine ligand 20 (CCL20) in both HEK293 and HT29 cells. IL-8 and CCL20 are well-known important regulators in NF-κB signaling and have been associated with chemoresistance in CRC and promote tumor progression [19]. NFKBIA is one of the most typical NF-κB target genes, thus, serving as an indicator of NF-κB activity. As shown in Figure 4E, there was a consistent trend with the RNA-seq data, indicating high ARMC4 expression downregulated the expression of NFKBIA, IL8, and CCL20 upon stimulation of IL-1β comparatively to the control in both HEK293 and HT29 cells. Collectively, these data suggest ARMC4 negatively regulates NF-κB signaling, decreases the expression of the majority of NF-κB target genes, and plays multiple roles in cell signaling networks.

### 2.3. ARMC4 Expression Decreases Cellular Proliferation, Migratory Ability, Anchorage-Independent Growth, and Tumor Growth

**Figure 5:**
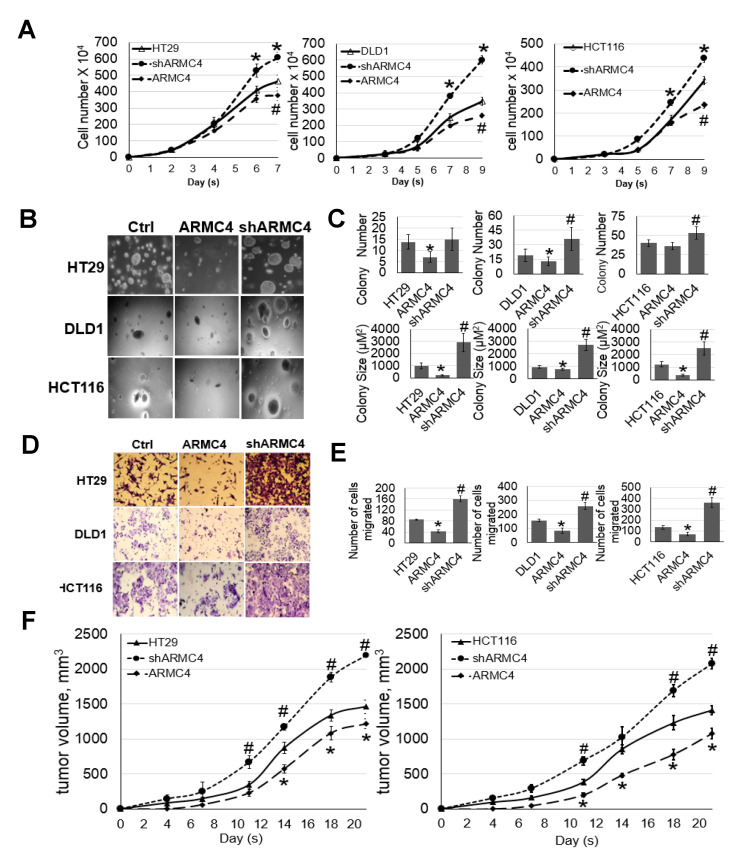
ARMC4 expression decreased cellular proliferation, migratory ability, anchorage-independent growth, and tumor growth. (**A**) Cell growth curve, showing that overexpression of ARMC4 inhibited cell growth compared with the Ctrl group, while shARMC4 knockdown greatly accelerated cell growth. * *p* < 0.05 ARMC4 vs. Ctrl group; # *p* < 0.05 shARMC4 vs. Ctrl group. (**B**,**C**) Anchorage-independent growth (Soft agar assay), showing ARMC4 overexpression cells exhibited dramatically decreased colony size and number compared with Ctrl cells. shARMC4 CRC cells showed increased colony size and number compared with Ctrl cells. * *p* < 0.05 ARMC4 vs. Ctrl group; # *p* < 0.05 shARMC4 vs. Ctrl group. (**D**,**E**) Migration assay using Boyden chamber wells, showing overexpression of ARMC4 decreased migratory ability of CRC cells compared to the Ctrl cells. In contrast, shARMC4 cells had remarkably increased migratory ability. * *p* < 0.05 ARMC4 vs. Ctrl group; # *p* < 0.05 shARMC4 vs. Ctrl. (**F**) Tumor growth of cells in an NSG mouse sub-cutaneous xenograft model, showing ARMC4 overexpression, led to decreased tumor growth compared to the Ctrl. shARMC4 led to significantly increased tumor growth compared to the Ctrl. * *p* < 0.05 ARMC4 vs. Ctrl group; # *p* < 0.05 shARMC4 vs. Ctrl group. *n* = 3 independent experiments for all experiments described here.

NF-κB target gene downregulation can indirectly or directly alter the cancer cell phenotype. Therefore, we need to determine ARMC4 expression’s impact on several of the known components of the cancerous phenotype regulated by NF-κB, including cell proliferation, migration, and tumor growth [20,21,22]. Firstly, we conducted cellular proliferation assays described in Figure 5A with high ARMC4 expression lines and knockdown stable cell lines described in Figure 3A. We observed that knockdown of ARMC4 significantly increased cellular proliferation while ARMC4 overexpression decreased cellular proliferation in CRC cells. We then wondered whether ARMC4 expression affected colony formation in an anchorage-independent environment. We carried out an anchorage-independent growth assay with soft agar using the same set of CRC stable cell lines in Figure 5B,C. ARMC4 overexpression decreased colony number and size compared to control cells, while shRNA knockdown of ARMC4 increased colony size and number, suggesting that ARMC4 negatively regulates colony formation in CRC cells. In addition to proliferative ability, the migratory ability of cancer cells is also critical for their uncontrolled spread. Using the same set of stable cell lines described above, we conducted Boyden chamber migration assays to determine the extent to which ARMC4 expression can affect migration of CRC cells. As shown in Figure 5D,E, shRNA knockdown of ARMC4 drastically increased cellular migratory ability. In contrast, overexpression of ARMC4 showed a marked opposite effect, confirming ARMC4’s role as a negative regulator for CRC cell migration. While these in vitro studies show several important aspects of ARMC4’s regulatory ability, we wished to further validate ARMC4’s role in a more clinically relevant sense by looking at tumor formation ability with high ARMC4 expression or shARMC4 knockdown in vivo. To conduct these studies, we used an NOD scid gamma (NSG) mouse model and subcutaneously implanted our stable or control cells of HT29 and HCT116 cell lines into mice. The volumes of tumor growth in the mice were determined until a set endpoint based on tumor size (≤2000 mm^3^). As shown in Figure 5F, the high ARMC4 expression in CRC cells decreased tumor growth while shRNA knockdown of ARMC4 significantly increased tumor growth. Together, these data suggest ARMC4 functions as a tumor suppressor in CRC.

### 2.4. ARMC4 May Function Downstream of IκB, and Complex with the p65 Subunit of NF-κB

While it is interesting to see that ARMC4 can negatively regulate NF-κB, we are keen to determine how ARMC4 regulates NF-κB activity. Firstly, we examined whether ARMC4 functions upstream or downstream of the step of IκBα degradation, which is important for NF-κB signaling. Stable CRC cell lines were treated with IL-1β for different time points, and Western blot assays were conducted to detect the level of IκBα. As shown in Supplementary Figure S1, no significant difference was observed among the high ARMC4 expressing or shRNA knockdown cells as compared to their control cells, indicating regulation of ARMC4 for NF-κB may function downstream of IκBα degradation.

**Figure 6:**
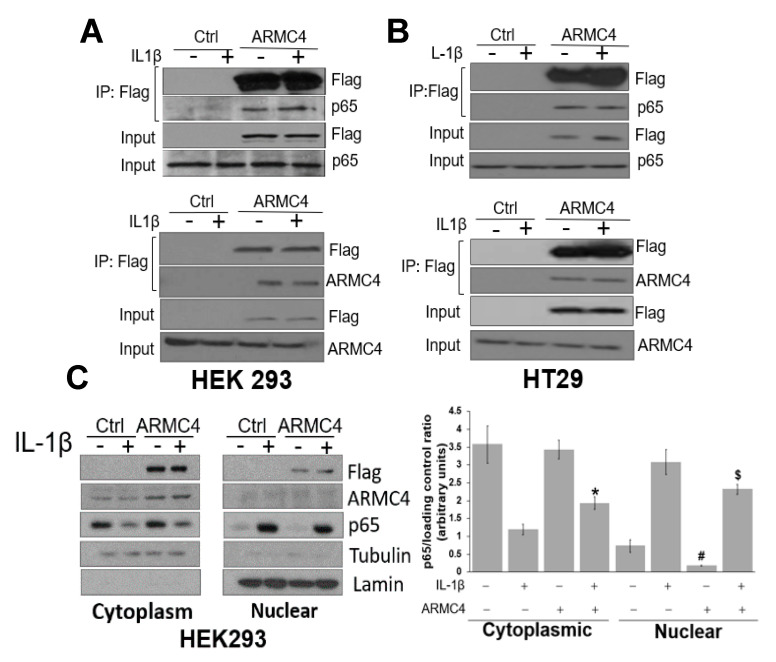
ARMC4 may complex with the p65 subunit of NF-κB. Co-IP experiments, showing ARMC4 may form a complex with the p65 subunit of NF-κB ((**A**,**B**) top panels). Co-IP experiments in HEK293 cells ((**A**) top panel) or HT29 cells ((**B**) top panel) were performed with or without co-expression of Flag-tagged ARMC4. Cells were treated or untreated with IL-1β for 1 h. Flag-ARMC4 was then pulled down with anti-Flag agarose beads. Samples were then subjected to Western analysis probed with anti-p65 antibody and anti-Flag antibody to detect the co-IP of ARMC4 and p65. For inputs, anti-Flag antibody was used to detect Flag-ARMC4; anti-p65 antibody was used to show the input of total p65. Similarly, co-IP experiments, showing p65 may form a complex with ARMC4 ((**A**,**B**) bottom panels). Co-IP experiments in HEK293 cells ((**A**) bottom panel) or HT29 cells ((**B**) bottom panel) were performed with or without co-expression of Flag-tagged p65. Experimental procedures are similar to those of top panels. For inputs, anti-Flag antibody was used to detect Flag-p65; anti-ARMC4 antibody was used to show the input of total ARMC4 (**C**) Cell fractionation experiment, showing localization of HEK293 cells control and overexpressing Flag-tagged ARMC4 cells to both the cytoplasm and nucleus. ARMC4 localized mostly to the cytoplasm, and ARMC4 overexpression reduced translocation of p65 to the nucleus. Tubulin and lamin were used as loading controls for cytoplasmic and nuclear fractions, respectively. ((**C**) right panel) Image J quantification of p65 to loading controls (tubulin and lamin) ratios for three independent Western blots is shown. * *p* < 0.05 cytoplasmic HEK293-ARMC4 + IL1β vs. cytoplasmic HEK293, # *p* < 0.05 nuclear HEK293-ARMC4 vs. nuclear HEK293, $ *p* < 0.05 nuclear HEK293-ARMC4 + IL1β vs. nuclear HEK293 + IL1β.

As mentioned previously, ARMC4 is known to play a critical role in binding and regulating protein interactions in PCD. Therefore, we speculate that ARMC4 may also engage in protein:protein interactions in the NF-κB pathway, leading to regulation of NF-κB signaling. To test this, we conducted co-IP experiments in both HEK293 and HT29 cells. As shown in Figure 6, the p65 subunit of NF-κB was pulled down together with ARMC4 in HEK293 (Figure 6A, top panel) or HT29 cell (Figure 6B, top panel) that overexpress Flag-tagged ARMC4, regardless of the presence of IL-1β. Similarly, reversed co-IP in either HEK293 (Figure 6A, bottom panel) or HT29 cells (Figure 6B, bottom panel) overexpressing Flag-tagged p65 showed similar results, confirming that ARMC4 and p65 could complex together or may interact together. 

During the process of NF-κB signaling, the p65 subunit translocates to the nucleus to bind to its cognate DNA elements. Logically, we would like to determine if ARMC4 and p65 co-localized to the same cellular compartments in cells during NF-κB signaling. We used Flag-tagged ARMC4 overexpressing HEK293 cells in cellular fractionation experiments in the presence or absence of IL-1β treatment to separate out the cytoplasmic and nuclear fractions. As shown in Figure 6C, HEK293 cells overexpressing Flag-tagged ARMC4 exhibited a mostly cytoplasmic localization as indicated by both Flag and total ARMC4 expression. Furthermore, IL-1β treatment stimulated the translocation of p65 to the nucleus in both HEK293 control and HEK293 ARMC4 overexpression cells as anticipated. However, we observed that in HEK293 ARMC4 cells, p65 expression was slightly decreased in the nucleus when compared to the HEK293 control cells upon stimulation of IL-1β (Figure 6C, left panel). This was quantified in three independent fractionation experiments via ImageJ software showing p65 expression was decreased in the nuclear fractions of HEK293 ARMC4 overexpression cells stimulated with IL-1β (Figure 6C, right panel). These data suggest there may be some interaction, sequestration, or other phenomena reducing the p65 translocation from the cytoplasm to the nucleus in HEK293 cells overexpressing ARMC4, allowing for NF-κB activity inhibition in these cells even when stimulated by IL-1β.

### 2.5. ARMC4 Expression Is Decreased in CRC

**Figure 7:**
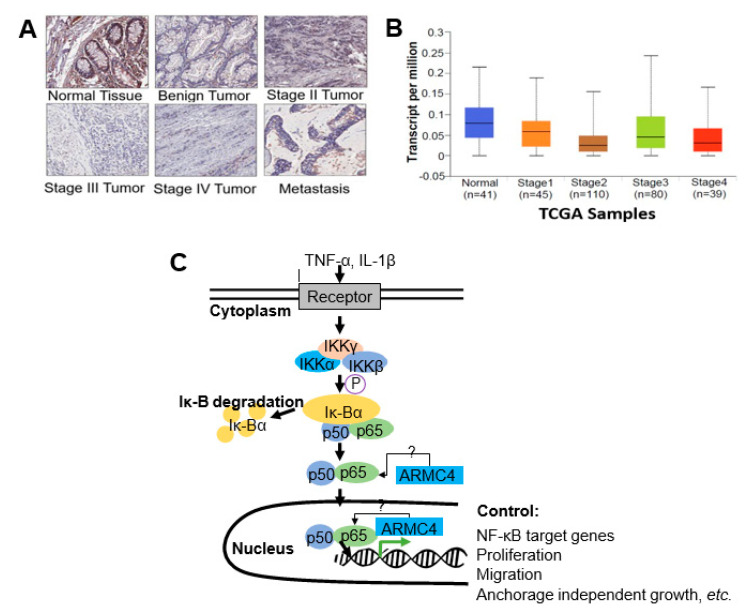
ARMC4 expression is decreased in CRC patient specimens. (**A**) Tissue microarray (TMA) using ARMC4 antibody as the probe, showing strong expression of ARMC4 in normal tissue, while significantly decreased expression in different stages of CRC and the metastasized patient tumor samples. (**B**) Data gathered from TCGA databases, showing ARMC4 transcripts trend toward decreased levels in different stages of CRC as compared to normal samples. (**C**) Hypothetical model, suggesting that ARMC4 may form a complex with the NF-κB, thus exert negative regulation effect on the hallmarks of cancer regulated by NF-κB. In traditional pathway, upon cytokine stimulation, the IκB kinase phosphorylates IκBα, causing degradation of IκB. The free p65/p50 heterodimer then migrates to the nucleus to bind to κB-binding sites on the promoters of specific genes, leading to their activation. Our findings suggest ARMC4 may complex with NF-κB downstream of IκBα, therefore decreasing NF-κB activity and reducing cellular proliferation, migration, and anchorage-independent growth ability as well as tumor growth in vivo.

Since we showed ARMC4’s novel role as a potential tumor suppressor in CRC, we wondered whether ARMC4 expression is correlated to CRC progression. We, therefore, conducted a tissue microarray (TMA) experiment using an ARMC4-specific antibody as a probe. As shown in Figure 7A, the ARMC4 expression is decreased in different stages of CRC versus the normal control. To look at this on a larger scale, we used TCGA databases, as shown in Figure 7B, which suggest a trend of decreased ARMC4 transcription in CRC as compared to the normal control. Statistical analysis for 7B samples can be found in Supplementary Figure S3. Collectively, these data suggest decreased ARMC4 expression may contribute to CRC progression in patients.

Collectively, we show that by using the highly powerful VBIM technique, we discovered ARMC4 as a previously unknown negative regulator of NF-κB. Our functional and mechanistic studies together lead us to a tentative hypothetical model (Figure 7C) by which we propose that ARMC4 inhibits NF-κB DNA binding and transactivation abilities downregulate the expression of NF-κB target genes, thereby inhibiting cell proliferation, anchorage-independent growth, migration, and tumor growth in NSG mice. We speculate this may occur through ARMC4’s complexing with NF-κB, potentially reducing the translocation of NF-κB into the nucleus. However, more details of these mechanisms remain to be discovered in the future.

## 3. Discussion

Our work here suggests a previously unknown role of ARMC4 in NF-κB signaling. ARMC4 is newly identified as a novel negative regulator of NF-κB using our VBIM technology (Figure 1 and Figure 2). High ARMC4 expression downregulated NF-κB target genes as well as had a marked effect on the regulation of gene networks (Figure 3 and Figure 4, and Supplementary Figure S2). Our data so far suggests ARMC4 may function as a tumor suppressor in CRC. We show that high ARMC4 expression decreased cellular proliferation, migration, and anchorage-independent growth. Additionally, high expression of ARMC4 decreased tumor growth in in vivo NSG mouse models (Figure 5). Mechanistically, not much is known about ARMC4’s functions in cancer. Our mechanistic studies suggest ARMC4 and the p65 subunit of NF-κB may complex together. Furthermore, as our cell fractionation studies show, ARMC4 and p65’s interactions may lead to possible sequestration or lagging of p65’s translocation to the nucleus (Figure 6). Furthermore, we have shown that ARMC4 functions downstream of the classical negative regulator of NF-κB activity, the step of IκBα degradation (Supplementary Figure S1). ARMC4 expression may also be decreased in CRC patient samples, supporting the notion that ARMC4 is a novel tumor suppressor in CRC (Figure 7).

While the ARMC4’s novel role in CRC may be clinically relevant, there are still several questions we aim to answer regarding future studies. One major logical question is if ARMC4 is mutated in CRC when compared to normal tissues. Searches of several CRC databases indicate a few missense mutations, which are fairly rare (data not shown). Interestingly, the mutations identified in CRC have never been associated with ARMC4’s known functions in PCD and mouse spermatogenesis.

Another main question we had was whether ARMC4 showed similarities to known tumor suppressors associated with CRC. For example, APC and p53, two well-characterized tumor suppressors, both have shown that their mutations could specifically drive CRC progression. Notably, p53 is mutated in about 70% of CRC patients [22]. Cooks et al. reported that mutant p53 can cause constitutive NF-κB activity, promote chronic inflammation, and subsequently drive further mutation in the progression of CRC [23,24]. Wild-type p53 acts to limit proliferation through transcriptional regulation of a cell’s life cycle by cell cycle arrest, senescence, and apoptosis [23,25]. Due to its critical cellular regulatory functions, mutations in p53 can lead to the promotion of cancer and are often seen in CRC [25]. Another famous tumor suppressor in CRC is APC, whose mutations cause dysregulation of the Wnt/β-catenin pathway, leading to CRC progression. APC is mutated in about 80% of all forms of CRC [26]. It controls the signaling of the Wnt/β-catenin pathway through cytoplasmic proteasomal degradation of β-catenin [27,28]. Loss of this function through mutated APC leads to disruption of apoptosis regulated by the Wnt/β-catenin pathway, leading to CRC progression [27]. It is noteworthy that while no links have been drawn between ARMC4 and its superfamily members APC and β-catenin, ARMC4 does share several major structural similarities with APC. The ARMC4 protein is made up of 10 tandem armadillo repeat motifs (ARMs), which form into ARM domains and one HEAT repeat, while APC contains 8 ARM domains and 4 coiled coil motifs. The ARM repeats are known to be important for transduction of Wnt signaling in embryonic development and are purported to form a superhelix that mediates protein-protein interactions [29]. Since both ARMC4 and APC have tandem ARM domains, it is possible that they can both form ARM superhelices, allowing them to bind other proteins. We speculate that the structural relationships between ARMC4 and APC suggest potential similar functional roles for ARMC4 and APC in binding proteins and possibly similar functions as tumor suppressors. However, these possibilities need to be further tested in future studies. While the data we have shown so far suggests several potential mechanisms of interaction between NF-κB and ARMC4, more details remain to be discovered. Of particular interest to us is the structure-function relationship between ARMC4 and APC. So far, we have shown full-length highly expressed ARMC4 decreases NF-κB transactivation activity. Additionally, our search of CRC patients sample databases indicates a low prevalence of mutations in ARMC4 in some CRC patients. Of further interest to us is the recent study by Liang et al., which indicates ARMC4 as a mutated gene in several patients with CRC ranging from stage I to stage III tumors with missense mutations [30]. This is consistent with our notion that ARMC4 functions as a tumor suppressor in CRC. In the future, we may generate truncated ARMC4 mutants to potentially disrupt the secondary structure of ARMC4 and its interactions with p65, thereby pinpointing ARMC4’s functional domains that are crucial to the regulation of NF-κB activity. Besides these avenues, an additional interesting aspect is the CRC microenvironment. We show that high ARMC4 expression in CRC cells produced the conditioned media that remarkably reduced NF-κB activity in stable 293-NF-κB reporter cells (Figure 3C). This phenomenon may be due to several reasons, such as ARMC4 downregulating target gene expression such as pro-inflammatory cytokines and thus their release into the microenvironment. To further test this hypothesis, we may pursue future studies such as cytokine array experiments for the CRC conditioned media.

Additionally, we show that ARMC4 has decreased expression in later stages of CRC (Figure 7), but the reasons behind this phenomenon are unknown. ARMC4 is known to have regulatory roles in PCD and mouse spermatogenesis, but what regulates ARMC4 itself is not known. In future work, we would like to identify regulators of ARMC4. This effort may help us uncover a novel therapeutic avenue for regulating constitutive NF-κB activity in CRC via control of ARMC4 expression. Besides its application as a potential therapeutic target, we may further pursue studies to determine if ARMC4 is a biomarker of CRC progression.

## 4. Materials and Methods

### 4.1. Cell Lines and Antibodies

The human embryonic kidney (HEK) 293 cells, also referred to as 293 cells, were cultured in Dulbecco’s modified Eagle’s media (DMEM) supplemented with 100 U/mL penicillin, 100 mg/mL streptomycin, and 10% fetal bovine serum (FBS). HEK293 cells were previously described [12]. The HT29, DLD1, and HCT116 colon cancer cell lines were purchased from the American Tissue Culture Collection (ATCC) (Manassas, VA, USA) and were cultivated in RPMI1640 media with 100 U/mL penicillin, 100 μg/mL streptomycin, and 10% fetal calf serum. Experiments were carried out when the cells reached 90% confluence. The following antibodies were obtained from commercial sources: anti-ARMC4, anti-NF-κB p65, and anti-IκBα were from Santa Cruz Biotechnology, Inc. (Dallas, TX, USA). Anti-β-actin and anti-Flag were from Sigma-Aldrich (St. Louis, MO, USA).

### 4.2. Virus Production, Cell Infection, and Selection

The VBIM and pLV-tTR-KRAB-Red lentiviruses were packaged in 293T cells using second-generation packaging constructs pCMV-dR8.74 and pMD2G (both packaging plasmids and pLV-tTR-KRAB-Red were kind gifts of Dr. Didier Trono, University of Geneva, Switzerland. Retroviruses encoding Cre-recombinase or empty vector control were packaged in Phoenix-Ampho cells. Supernatant media-containing virus, collected at 36–48 h, were supplemented with 4 µg/mL polybrene before being frozen in aliquots. To perform selections in Z3 cells, infections were performed so that 70–90% of each population was GFP-positive before selection with drugs. Z3 cells, pretreated with 25 μg/mL of zeocin for 7 days to remove any background mutants, were cultured at 1 × 10^5^ cells/well, into 30 wells of 12-well plates. The next day, the cells were infected with the three different VBIM viruses (SD1, 2, and 3). The medium was replaced 24 h after infection, and the cells from each well were split and transferred into a 15 cm plate 48 h later. After another 24 h, medium containing 0.1 μg/mL of GCV was added and replaced every three days. Individual clones were picked after 2 weeks.

### 4.3. Construction of Stable ARMC4 Overexpressing or shARMC4 Cells

ARMC4 gene was cloned into the lentiviral vector as a full-length cDNA or shRNA pool (containing five different shRNA constructs) against ARMC4 were purchased from Sigma-Aldrich (St. Louis, MO, USA). Viruses were generated and used to infect HT29, HCT116, DLD1, and HEK293 cells. Since both the lentiviral vector and the shRNA pool carry the puromycin resistance marker, puromycin-resistant clones were selected in 1 μg/mL puromycin after virus infection. Then cells were pooled and tested for the best overexpression and knockdown by the Western blot method with antibodies against ARMC4.

### 4.4. Transfections and Luciferase Assays

For NF-κB luciferase assays, the κB-luciferase construct p5XIP10 κB (contains five tandem copies of the NF-κB site from the IP10 gene) was transfected transiently into the cells, and luciferase activity was assayed 48 h later. A β-galactosidase (β-gal) construct was co-transfected to normalize for transfection efficiency. The cells were then washed with cold phosphate-buffered saline (PBS) and lysed in 80 μL of 5X lysis buffer from Promega Corporation (Madison, WI, USA). After incubation on ice for 15–20 min, cell debris was pelleted at 15,000× *g* for 5 min at 4 °C. A measure of either 30 μL of luciferase assay substrate or β-gal substrate (Promega Corporation) was added to 20 μL of supernatant solution before being read in a luminometer. The relative luminescence was normalized to the optical reading of β-gal (Bio-Rad Laboratories, Hercules, CA, USA). Luciferase activity was measured using a Synergy H1 Multi-Mode Reader (BioTek Instruments Inc., Winooski, VT, USA). 

### 4.5. PCR, Quantitative PCR

The total RNA from mutant cells was extracted with the TRIzol^®^ reagent at room temperature following the protocol from manufacturer (Invitrogen Life Technologies, Waltham, MA, USA). Reverse transcription was performed using oligo DT20 primers with the SuperScript III First-Strand Synthesis System kit and protocol (Invitrogen Life Technologies, Waltham, MA, USA). Standard PCR reactions were further performed by using a forward primer from the VBIM vector [9] and Oligo dT primer from the SuperScript III First-Strand Synthesis System kit, and EconoTaqPLUS Green 2X PCR Master Mix (Lucigen, Middleton, WI, USA). The PCR product was directly cloned into the pCR2.1 vector (Invitrogen Life Technologies, Waltham, MA, USA). The plasmid was amplified and sent for sequencing. For qPCR experiments, cells were cultured to 80–90% confluency and were untreated or treated with IL-1β for 4 h. RNA was collected and purified as described above, and first-strand cDNA was synthesized by SuperScript III as described above. Samples were further analyzed by using FastStart Universal SYBR Green Master (ROX) (Roche Diagnostics, Indianapolis, IN, USA) qPCR reactions. The quantitative mRNA expression was calculated based on the 2^−ΔΔCT^ method [20]. GAPDH was selected as the housekeeping gene for normalization; each gene was running along with GAPDH, and the difference between threshold cycles (CT) was designated as ΔCT. ΔΔCT is the difference between their respective controls. PCRs were run with the 7500 Real Time PCR System (96-well format) (Applied Biosystems., Salt Lake City, UT, USA). IL8-Forward: 5′-TCCTGATTTCTGCAGCTCTGT-3′; IL8-Reverse: 5′-AAATTTGGGGTGGAAAGGTT-3′; CCL20-Forward: 5′-GTGCTGCTACTCCACCTCTG-3′; CCL20-Reverse: 5′-CGTGTGAAGCCCACAATAAA-3′; NFKBIA-Forward: 5′-CCCAAGCACCCGGATACAG-3′; NFKBIA-Reverse: 5′-CATAGCTCTCCTCATCCTCACTCTCT-3′.

### 4.6. Western Analysis

Cells were cultured to 95% confluency, treated or untreated with IL-1β at different times, as indicated in the descriptions of individual experiments and washed with 1X PBS, and pelleted at 5000× *g* at 4 °C for 4 min. Cell pellets were lysed with RIPA buffer (1X PBS/1% Nonidet p-40/0.5% sodium deoxycholate/0.1% sodium dodecyl sulfate (SDS)). Cellular debris was removed by centrifugation at 15,000× *g* for 10 min. The amount of protein in the supernatant solution was determined, and samples were heat-treated in 2X SDS sample loading buffer at 100 °C for 5 min. Equal amounts of samples were fractioned by SDS/PAGE and transferred to nitrocellulose membranes. Membranes were blocked in 5% non-fat skim milk powder in PBS for an hour and then probed with primary antibodies, which were visualized with horseradish peroxidase-coupled secondary antibodies by using the Enhanced Chemiluminescence (ECL) Western Blotting Detection System (PerkinElmer Life and Analytical Sciences, Waltham, MA, USA). Human IL-1β (Pepro-Tech., East Windsor, NJ, USA) was used at 10 ng/mL.

### 4.7. Co-Immunoprecipitation Assay

Cells cultured in 10 cm plates to 95% confluency were lysed in co-immunoprecipitation buffer. Cells were then lysed with co-immunoprecipitation buffer (1% Triton X-100, 50 mM Tris-HCl, pH 7.4, 150 mM NaCl, 1 mM EDTA, 1 mM sodium orthovanadate, 20 μM aprotinin, and 1 mM phenylmethanesulfonyl fluoride and pepstatin A). After spinning the debris for 10 min at 4 °C, the supernatant solution was incubated with EZview Red anti-Flag M2 affinity gel (Sigma-Aldrich, Burlington, MA, USA) overnight at 4 °C. Gel beads were washed with 20 volumes of immunoprecipitation buffer, with rotation at 4 °C for 5 min each time. Protein was eluted with Flag peptide (Sigma-Aldrich, Burlington, MA, USA), following Sigma’s standard protocol. The supernatant solution was mixed with 5XSDS sample loading buffer, boiled for 5 min, and separated in a 10% Tris-HCl SDS/PAGE gel.

### 4.8. Cell Proliferation and Anchorage-Independent Growth Assays

HT29, DLD1, or HCT116 control cells or cells with overexpression or shRNA knockdown of ARMC4 were plated in triplicates at 20,000/well in a 6-well plate with 3 mL of medium, and the medium was changed every 3 days. Cell number was counted on different days using a cell counting chamber. For anchorage-independent growth assays, type VII agarose (Sigma-Aldrich, Burlington, MA, USA) was autoclaved and mixed with RPMI1640 cell growth medium. Cell culture dishes were coated with 1.2% agarose as the bottom layer. Cells were resuspended in 0.6% of soft agarose and plated on top of the bottom layers. Cells were cultured in the semisolid medium for about 2–3 weeks. The colonies formed were checked under a microscope and measured and counted with the help of ImageJ software (ver. 1.47t).

### 4.9. Migration Assay

The Boyden chamber containing cell culture inserts with polycarbonate membrane at the bottom with an 8 μM pore size and 6.5 mm diameter (Corning-Costar, Lowell, MA, USA) were used. HT29, DLD1, or HCT116 control cells, or cells with the overexpression or shRNA knockdown of ARMC4 were suspended in serum-free RPMI1640 medium and plated in triplicates at 200,000/insert in the upper chambers. The lower chambers were filled with 10% FBS RPMI1640 medium. The cells were incubated at 37 °C for 72 h. The inserts were then removed, and migratory cells at the bottom of the chamber were fixed and stained in a 4% paraformaldehyde (PFA)/0.1% Crystal Violet solution, followed by washing in deionized water to remove redundant staining. Non-migrated cells remaining at the upper side of the membranes were carefully removed with cotton swabs, and inserts were dried in darkness overnight. The following day stained membranes were pictured in five random non-overlapping fields and counted manually at 20× objective and 20× eyepiece on a transmitted-light microscope.

### 4.10. Immunohistochemical (IHC) Analysis of Tissue Microarray (TMA)

The colon cancer TMA CO951 (30 cases/95 cores) was purchased from US Biomax, Inc. (Rockville, MD, USA). The cores were duplicated, 30 cancer and 8 of which have matched normal adjacent tissue and 10 cases of matched metastasis, with follow-up data 2 cores per case. All histochemical stains were carried out at the Indiana University School of Medicine IHC Core, using standard procedures included standard deparaffinization in xylene, quenching in 1% hydrogen peroxide/methanol for 10 min, and rehydrated through sequentially graded ethanol. Antigen retrieval was performed by ethylenediamine-tetraacetic acid (EDTA). Using DAKO automated immunostrainers (DAKO, Carpinteria, CA, USA), the slides were blocked for 30 min in horse serum and incubated with ARMC4 antibody, followed by incubation with secondary antibody. The Universal ABC Elite kit (Vectastain, Burlingame, CA, USA) with 3,3′-diaminobenzidine development was used to visualize antibody binding, and the slides were subsequently counterstained with hematoxylin. The tissue arrays were stored at 4 °C and heated to 60 °C for 1 h before use.

### 4.11. Evaluation of IHC Staining

The TMA sets were scanned using Images were scanned with the Aperio Scanscope Imaging System (Leica Biosystems, Buffalo Grove, IL, USA). The TMA tissue cores were individually copied, labeled, and stored in a separate folder as tiff files. The IHC staining results of individual core tissues were evaluated by an expert pathologist, who was blinded to the patient’s clinocopathological details. The IHC staining was categorized according to a scoring method based on the staining intensity (score 0, no staining intensity; score 1, weak staining intensity; score 2, intermediate staining intensity; and score 3, strong staining intensity). In the case of heterogeneous staining within the samples, the respective higher score was chosen if >50% of the cells showed higher staining intensity.

### 4.12. Conditioned Media Assay

The HT29, DLD1, and HCT116 cells were seeded into 12-well plates, cultivated to 90% confluency, and transfected with different plasmids: empty vector, ARMC4, shARMC4. After 24 h of transfection, the media were replaced, and the cells were kept for an additional 48 h. The conditioned media were collected, floating cells were pelleted at 3000× *g* at 4 °C, for 10 min, and the supernatant was aliquoted into sterile tubes and either used immediately or stored at −80 °C. The media were then used to treat 293-NF-κB reporter cells, and luciferase assay was performed as previously described. Detailed procedures have been described by Lu et al., 2009.

### 4.13. RNA-Seq Analysis

RNA-seq analysis was carried out at the University of Chicago Genomics Facilities. Ultrapure RNAs were prepared essentially described previously [20]. Briefly, cells were cultured to ~90% confluence, and total RNA was isolated using TRIzol^®^ reagent. RNA samples were further processed by the University of Chicago Genomic Facilities for the RNA-seq experiment.

### 4.14. Ingenuity Pathway Analysis (IPA)

Groups of genes were analyzed by the IPA software11. The setting and filter were as follows: reference set: Ingenuity Knowledge Base (Genes_Endogenous Chemicals); Relationship to include: Direct and Indirect; Includes Endogenous Chemicals; Filter Summary: Consider only molecules where species_Human OR Rat OR Mouse. The *p* values for the enrichment test were calculated using Fisher’s exact test, right-tailed. Log10 (*p*) was visualized to the left of the *p*-value. *p* < 0.05 was considered to be statistically significant.

### 4.15. Statistical Analysis

The data represent the means ± SEM from at least three separate experiments performed in triplicate. The differences between groups were analyzed using Student’s *t*-test, and a *p*-value < 0.05 was considered statistically significant. Statistical analyses were carried out using JMP software (ver. 7.0).

## 5. Conclusions

In summary, our study has uncovered ARMC4 as a novel negative regulator of NF-κB and a tumor suppressor in CRC. NF-κB signaling is hyperactive in many cancers, including CRC; thus, discovery of its novel negative regulator, identifying ARMC4’s innovative role in CRC regulation, and delineating an exciting new avenue for a potential therapeutic approach to CRC holds great significance in the CRC research field. Prior to our work, ARMC4 has only been shown to have roles in the areas of PCD and mouse spermatogenesis with a limited focus on the mechanisms of action of ARMC4 [16,17,18]. In the context of those diseases, specific mutations caused loss of motor function. Here we describe ARMC4’s novel role in cancer through NF-κB signaling. The studies here and future work may help elucidate the complexities of the NF-κB signaling network in CRC. Furthermore, since ARMC4 expression is also decreased in several other cancers, including breast and ovarian cancers, our identification and characterization of ARMC4’s function in CRC may serve as the basis for future studies into other hyperactivated NF-κB related cancers or diseases as well.

## Data Availability

Data available upon request to authors.

## Supplementary Materials

The following supporting information can be downloaded at: https://www.mdpi.com/article/10.3390/ijms23052732/s1.
