# Supplementary material for: Using VBIM Technique to Discover ARMC4/ODAD2 as a Novel Negative Regulator of NF-κB and a New Tumor Suppressor in Colorectal Cancer
Source: Int J Mol Sci. 2022 Mar 1;23(5):2732. doi: 10.3390/ijms23052732 (PMC8910849; doi:10.3390/ijms23052732)
Supplement: Supplementary file 1 [file ijms-23-02732-s001.zip › ijms-1610169-supplementary.pdf]

Supplementary Information

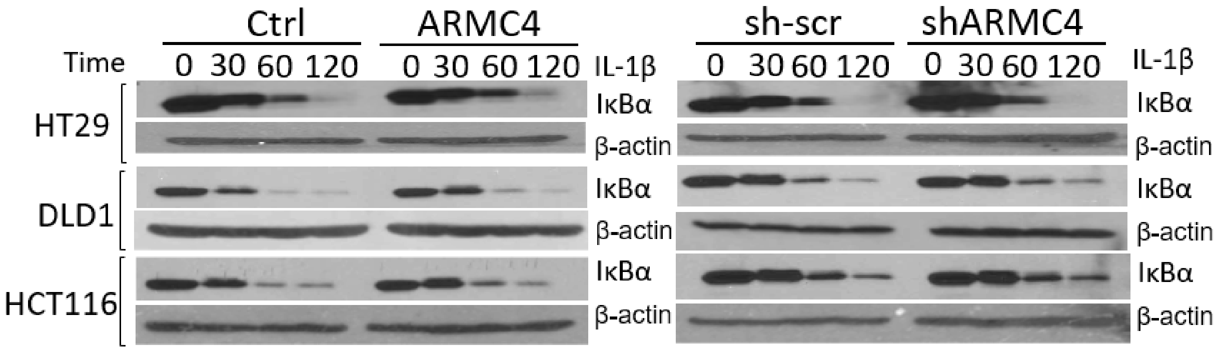

Supplementary Figure S1. IκBα degradation is similar in ARMC4 knockdown, overexpression, and control lines. Western Blot, showing IκBα expression is similar in CRC cell lines HT29, HCT116 and DLD1 in overexpression, knockdown and shscramble lines.

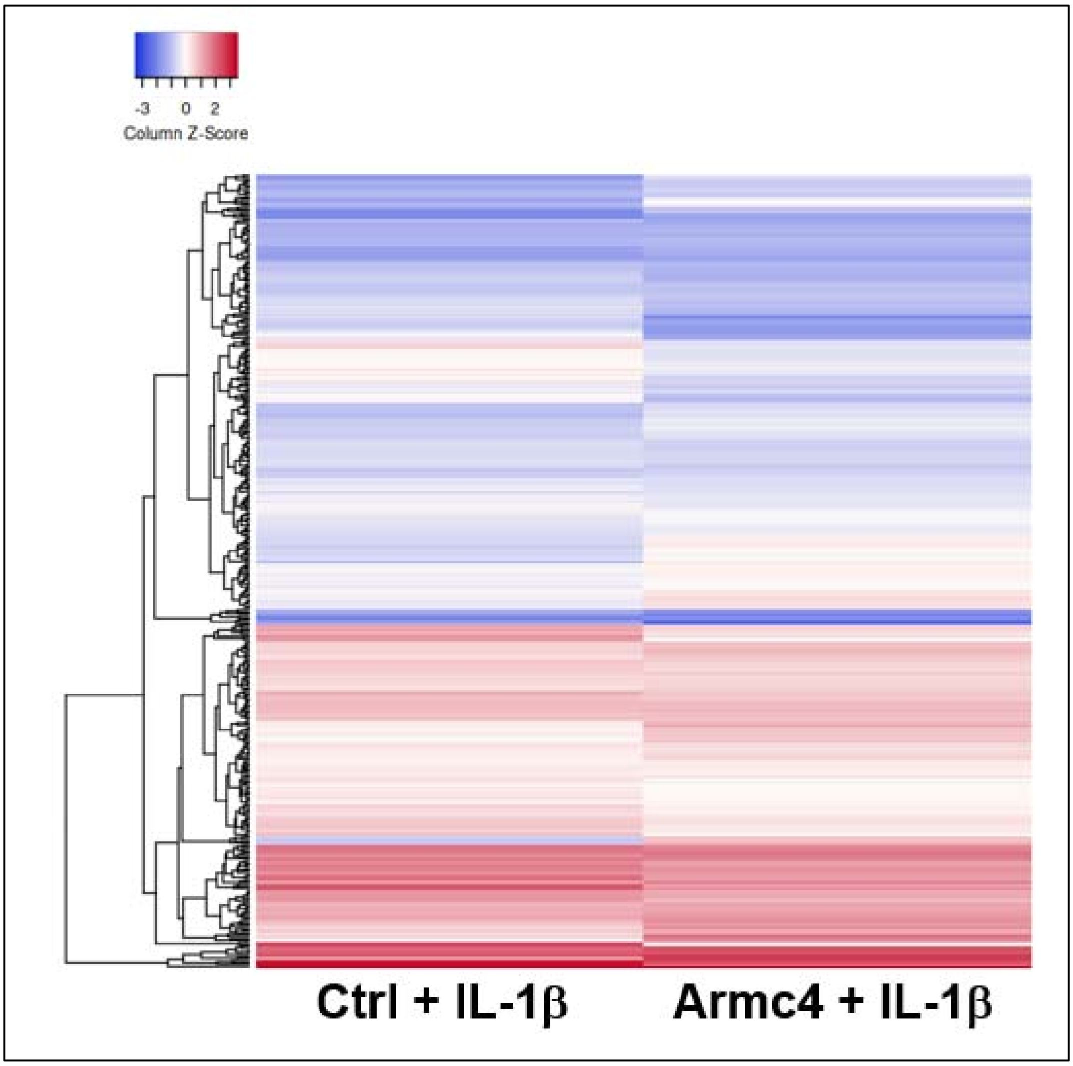

Supplementary Figure S2. Heatmap, with normalized expression data (log transformed FPKM) for NF-κB-inducible genes (398 genes, based on Ctrl+IL-1β vs. Ctrl ≥ 6). Genes were clustered using average linkage method. Software used: http://www.heatmapper.ca/expression/

Comparison	Statistical significance
Normal-vs-Stage1	4.518400E-01
Normal-vs-Stage2	2.060500E-02
Normal-vs-Stage3	1.992440E-01
Normal-vs-Stage4	2.579600E-01
Stage1-vs-Stage2	9.704100E-02
Stage1-vs-Stage3	6.477400E-01
Stage1-vs-Stage4	6.833600E-01
Stage2-vs-Stage3	4.256000E-02
Stage2-vs-Stage4	2.362200E-01
Stage3-vs-Stage4	9.602800E-01

Supplementary Figure S3. Statistical analysis of Figure 7B. Staging comparisons versus normal and between stages. Statistics provided on http://ualcan.path.uab.edu/cgi-bin/TCGAExResultNew2.pl?genenam=ARMC4&ctype=COAD.
